# Many faces, one rule: the role of perceptual expertise in infants’ sequential rule learning

**DOI:** 10.3389/fpsyg.2015.01595

**Published:** 2015-10-21

**Authors:** Hermann Bulf, Viola Brenna, Eloisa Valenza, Scott P. Johnson, Chiara Turati

**Affiliations:** ^1^Dipartimento di Psicologia, University of Milano-BicoccaMilano, Italy; ^2^Milan Center of Neuroscience (NeuroMI)Milano, Italy; ^3^Dipartimento di Psicologia dello Sviluppo e della Socializzazione, Università degli Studi di PadovaPadova, Italy; ^4^Interdepartmental Center for Cognitive Science, Università degli Studi di PadovaPadova, Italy; ^5^Department of Psychology, University of CaliforniaLos Angeles, Los Angeles, CA, USA

**Keywords:** rule learning, face, infants, inversion effect, perceptual expertise

## Abstract

Rule learning is a mechanism that allows infants to recognize and generalize rule-like patterns, such as ABB or ABA. Although infants are better at learning rules from speech vs. non-speech, rule learning can be applied also to frequently experienced visual stimuli, suggesting that perceptual expertise with material to be learned is critical in enhancing rule learning abilities. Yet infants’ rule learning has never been investigated using one of the most commonly experienced visual stimulus category available in infants’ environment, i.e., faces. Here, we investigate 7-month-olds’ ability to extract rule-like patterns from sequences composed of upright faces and compared their results to those of infants who viewed inverted faces, which presumably are encountered far less frequently than upright faces. Infants were habituated with face triads in either an ABA or ABB condition followed by a test phase with ABA and ABB triads composed of faces that differed from those showed during habituation. When upright faces were used, infants generalized the pattern presented during habituation to include the new face identities showed during testing, but when inverted faces were presented, infants failed to extract the rule. This finding supports the idea that perceptual expertise can modulate 7-month-olds’ abilities to detect rule-like patterns.

## Introduction

A central question in developmental research concerns how infants learn to detect relations between different elements and to generalize these relations to new elements that may have no surface features in common to those previously encountered. This learning mechanism, known as rule learning, is crucial to extraction of structure from our environment and its consistencies across space and time.

Rule learning was first investigated in the linguistic domain by Marcus et al. (1999). The authors assessed infants’ ability to extract rules from a speech sequence, familiarizing 7 months old infants to sequences of syllables that followed a particular grammar (e.g., *la ta ta, gai mu mu*, which is ABB). Given 2 min of exposure, infants were able to discriminate between novel sequences following the same pattern (e.g., wo fe fe, for ABB), and novel sequences following a different pattern (e.g., *wo fe wo*, which is ABA). The test syllables had not been used in training, suggesting that infants can extract a rule, generalize it to novel stimuli that may have no surface features in common with those presented in training, and discriminate it from other, similar patterns. In contrast to their success in learning rules from speech, 7-month-olds failed to learn rules from non-linguistic auditory stimuli, including animal sounds, pure tones, notes of different timbre (Marcus et al., 2007), and chords (Dawson and Gerken, 2009). These findings could be taken to suggest that rule learning is a mechanism specific for language acquisition, innately predisposed to process speech sounds (Marcus et al., 1999, 2007).

However, a number of studies have cast doubt on this claim. For example, using near-infrared spectroscopy, it has been found that newborns did not exhibit a rule-learning mechanism when they were exposed to linguistic sequences of syllables (Gervain et al., 2008). Newborn babies were able to detect adjacent repetitions such ABB, but they failed in detecting non-adjacent repetitions such as ABA, providing evidence that at birth infants may have a domain-general perceptual “repetition detector,” instead of a true ability to extract rule-like patterns. Moreover, 4-month-old infants, but not 7-month-olds, learned rules from non-linguistic auditory stimuli, such as sequences of musical chords or tones, suggesting that 7-month-olds’ rule learning would have been tuned to the linguistic domain as a consequence of the different experience with language and music acquired between 4 and 7 months of age (Dawson and Gerken, 2009). These lines of evidence appear to contradict the account that rule learning is an innate mechanism specific for speech processing.

The claim that rule learning is not specific to language acquisition is supported by recent studies that have investigated infants’ ability to detect and generalize rules from visual stimuli. Saffran et al. (2007) have demonstrated that 7 months old infants can learn sequential rules from visual stimuli that they can readily represent and categorize, such as images of dogs or cats. The authors argued that, instead of being evolved to subserve language learning, rule learning can be considered as a more general mechanism that is modulated by the familiarity and the categorizability of the stimuli to be learned: familiar stimuli, no matter whether they belong to linguistic or visual domains, enhance infants’ ability to detect and generalize rules. This idea is supported by a recent study that has investigated 8- and 11-month-olds’ rule learning abilities in the presence of unfamiliar visual shapes (Johnson et al., 2009). Indeed, when visual stimuli are unfamiliar, infants’ rule learning abilities are weaker than with familiar stimuli. For instance, 7.5 months old infants’ ability to extract rules from a sequence of communicative but unfamiliar sign language-like gestures is limited to some patterns (i.e., ABB vs ABA, but not the reverse) (Rabagliati et al., 2012). Also, 5-month-old infants learn rules that are jointly instantiated in shapes and syllables, but not rules from shapes alone (Frank et al., 2009). Overall, these findings suggest that rule learning can be applied also to visual stimuli, and it is facilitated when the information presented is highly familiar to infants.

Yet, quite surprisingly, to our knowledge, infants’ ability to detect rules has never been investigated using a salient stimulus category for which infants commonly accumulate an extensive visual experience, such as faces. Faces are the stimuli that we likely encounter more often in the visual environment from early in life, being an important medium for the child’s cognitive, emotional, and social development. Indeed, newborns preferentially attend to faces (Valenza et al., 1996; Macchi Cassia et al., 2004) and show surprisingly refined face discrimination capacities (e.g., Pascalis and de Schonen, 1994; Turati et al., 2006, 2008).

Faces convey a great amount of visual information, both transitional (i.e., emotions, gaze direction) and stable (i.e., species, race, gender), and face recognition is spontaneously, efficiently, and routinely performed by humans. This specialization in face processing is explained as a result of the perceptual expertise with this category of stimuli acquired through development (e.g., Diamond and Carey, 1986; Tarr and Gauthier, 2000; Gauthier and Nelson, 2001). Furthermore, there is mounting evidence that perceptual experience has a critical role in building face representation even in the first months after birth. For example, during the first year after birth, infants’ face-processing skills tune around faces of the most experienced species (Pascalis et al., 2002; Di Giorgio et al., 2012), race (Kelly et al., 2007, 2009), and age (Macchi Cassia et al., 2014), providing evidence that the amount of early environmental exposure to different face types shapes infants’ face processing abilities. Although a long developmental time course is required in order to achieve the adult level of expertise at discriminating individual faces, key aspects of adult face recognition (e.g., sensitivity to configural cues) develop in the first years after birth and, in many cases, in infancy (McKone et al., 2012). One traditional example of configural processing is the *inversion effect*, which refers to the disproportionate drop in performance for face recognition relative to object recognition due to stimulus inversion (Yin, 1969). Recent evidence has shown that infants process faces differently according to whether the stimuli are presented upright or inverted, providing evidence for the presence of an inversion effect during early infancy (e.g., Turati et al., 2004). Turati et al. (2004) showed that, under some experimental conditions, 4-month-olds’ face recognition abilities are limited by stimulus orientation. This orientation difference in infants’ face processing was confirmed by an eye movement study showing qualitative differences in the way that 4-month-olds explored upright and inverted faces (Gallay et al., 2006). Overall, these studies provide evidence for a crucial role of perceptual expertise in shaping face-processing skills during the first months after birth, rendering the use of faces particularly suitable in investigations of whether and how rule learning might be affected by stimulus familiarity at early stages of development.

The aim of the present study was to investigate 7-month-olds’ ability to recognize and generalize rule-like patterns when constituent elements of the patterns are faces. Moreover, we examined the role of perceptual experience in 7 months old infants’ ability to detect rule-like patterns by presenting infants with sequences of images of upright and inverted faces. The images of inverted faces were identical to the images of upright faces except for the orientation. To our knowledge this is the first time in which infants’ rule learning abilities were investigated by directly comparing a frequently experienced category of visual stimuli with an infrequently experienced category.

Using an infant-controlled visual habituation paradigm, 7-month-olds were habituated to triads of faces in an ABA condition (i.e., a face A was followed by a different face B, that was in turn followed by the face A), or in an ABB condition. In the test phase, ABA and ABB triads, composed by faces that differed from those showed during habituation, were presented. If infants learned the rule inherent in the face triplets presented during habituation and generalized it to the new face identities, they should look longer at the novel rule as compared to the familiar one during the test phase. Infants were presented with an upright condition, in which face triplets were composed of upright faces, or with an upside-down condition, in which faces triplets were composed of inverted (upside-down) faces. We expected that upright faces, in contrast to inverted faces, would be treated as familiar stimuli by 7 months old infants, leading to an advantage in the rule-learning task compared to inverted faces.

## Materials and Methods

### Participants

Seventy-one 7 months old infants (35 females, *M*_age_ = 225 days; range = 209–244 days) were included in the final analyses. All participants were healthy and full-term, and they were all Caucasian. Twenty-three additional infants were excluded from the final sample because of fussiness (*N* = 19), preterm birth (*N* = 2), or medical problems in the first months (*N* = 2). Participants were recruited via a written invitation that was sent to parents based on birth record provided by neighboring cities. Infants were randomly assigned to the upright condition (*N* = 35) or to the inverted condition (*N* = 36), in which face sequences were composed by upright and inverted faces, respectively. The procedure was approved by the University Ethical Committee. Parents gave written informed consent for their infants’ participation.

### Stimuli

**FIGURE 1:**
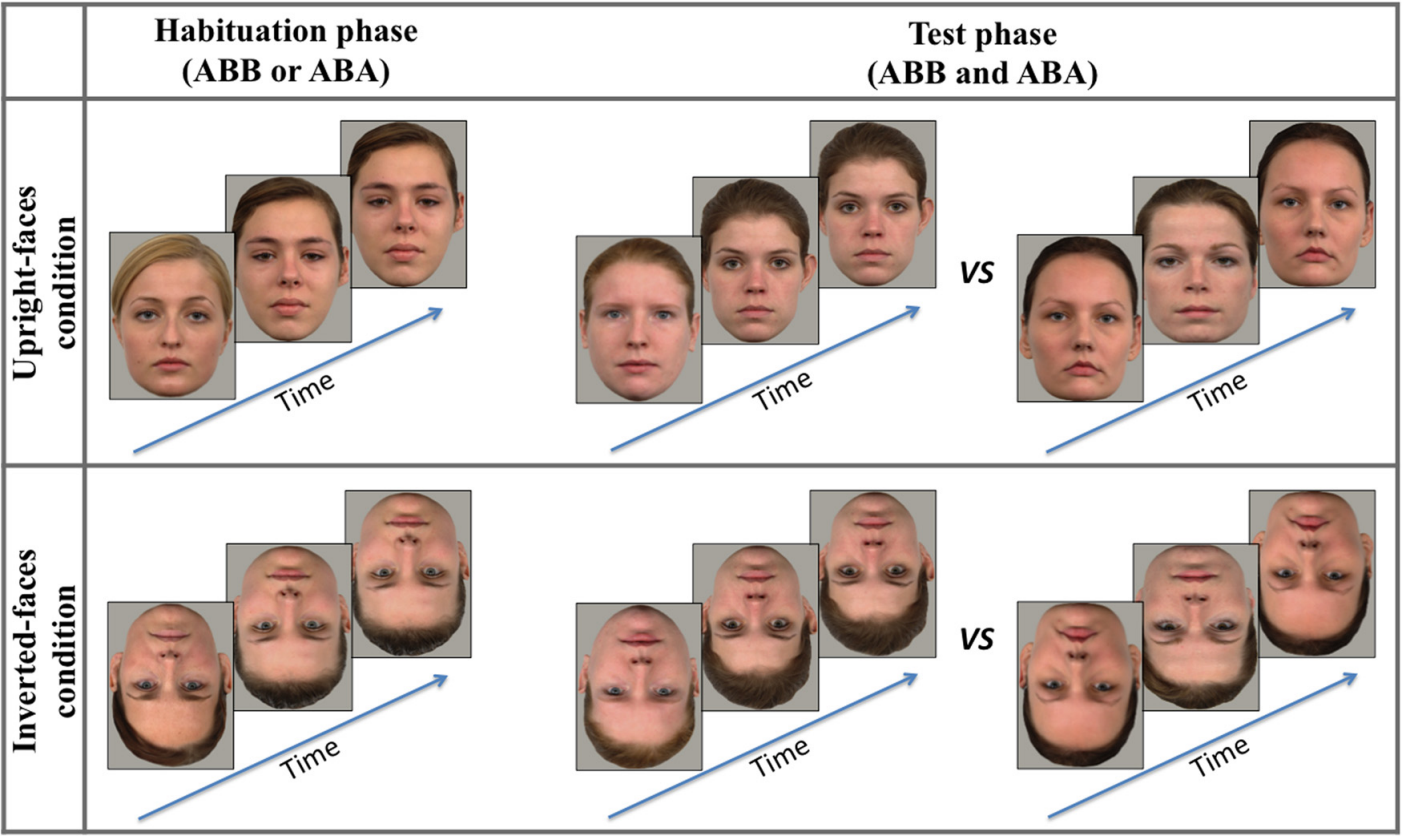
**Examples of the stimuli used during the habituation and test phases**.

Upright faces were color photographs of 12 female adult faces of Caucasian origin, all displaying a full-front neutral expression with open eyes. The images were taken from the Radboud Faces Database (Langner et al., 2010). Using the software Adobe Photoshop, face images were cropped maintaining some external features like ears and hair, and pasted on a gray background (**Figure 1**). When viewed from approximately 60 cm, adult faces measured 19° in height and 14° in width. Inverted faces were the same 12 female faces turned upside-down by 180°.

### Apparatus

All infants were tested in a dedicated cabin, while seated in an infant-seat or on the parent’s lap and positioned at a distance of approximately 60 cm from a 61-cm computer screen. The whole experiment was recorded through a video-camera, hidden over the screen, which fed into a TV monitor and a digital video recorder, both located outside the testing cabin. The TV monitor displayed the live image of the infant’s face to allow the online coding of the infant’s looking times through the E-Prime program by the experimenter, who was outside the testing cabin and blind to the condition to which the infant was assigned. The image of the infant’s face was also recorded via a Mini-DV digital recorder for a frame-by-frame oﬄine coding of looking times during test trials.

### Procedure

We adopted the general procedure used by Saffran et al. (2007). Eight different face identities were used to create the habituation triads, and four different face identities were used to create the test triads. For the habituation sequences, four face identities were assigned to the A group and four to the B group. The A and B images were randomly combined by the software to generate 16 different ABA triads (i.e., a face A was followed by a different face B that was in turn followed by the face A) and 16 different ABB triads. For the test sequences, triads were made up of four novel face identities, two assigned to the group A and two assigned to the group B.

A left-to-right sequential and simultaneous presentation of the face images within each triad was used. The first picture was displayed alone for 330 ms, toward the left edge of the screen. The second picture was then added in the middle of the monitor, to the right of the first picture; this two-face display was presented for 330 ms. Then the third picture was added, to the right of the second picture, and the full triad was displayed for 830 ms, for a total of 1.5 s for each triad. A blank screen (500 ms) separated the triad presentations on each trial. In each condition (upright and inverted), half of the infants was randomly assigned to the ABB habituation condition, the other half to the ABA habituation condition (**Figure 1**).

An infant-controlled habituation procedure was used. Testing began with a central cartoon animated image associated to a sound to catch infants’ attention. As soon as the infant fixated the screen the experimenter turned off the cartoon and activated a trial, so that the habituation phase began. Each trial consisted of triads of faces, presented in a random order, organized in either the ABB or ABA pattern. The experimenter recorded infant’s fixation by holding the mouse button whenever the infant fixated on the stimulus. If the infant looked away from the stimulus for more than 2 s, the trial ended and a cartoon animation reappeared on the screen to re-attract the infant’s attention before a new trial was presented. The habituation phase ended when the sum of infant’s looking times on three consecutive trials was equal to or less than 50% of the total looking time from the infant’s first three trials (Slater et al., 1985). When this habituation criterion was reached, the stimulus was automatically turned off and a new cartoon animation image was turned on. As soon as the infant’s gaze was realigned to the animation, the test phase began. All infants received the same set of six test trials in which ABA and ABB triads, composed by faces that differed from those showed during habituation, were presented alternately, each for three times. The order of presentation (i.e., novel or familiar first) was counterbalanced among infants.

Means of looking times (s) toward novel or familiar pattern were considered as the dependent variable. About one third of the infants (*N* = 20) was coded oﬄine by a second independent observer who was blind to the experimental conditions. Inter-observer agreement (Pearson correlation) between the two observers (i.e., the one who coded the data online and the one who coded from digital recording), as computed on total fixation times during test trials, was *r* = 0.97.

## Results

**FIGURE 2:**
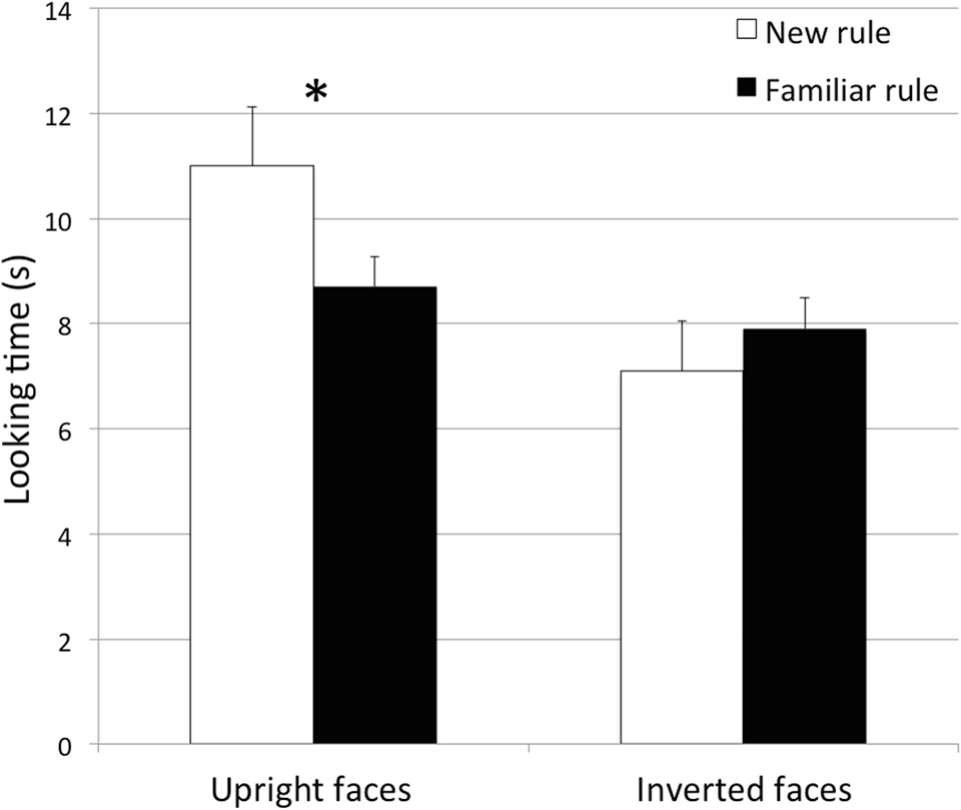
**Looking times after habituation.** Infants looked longer at the novel rule than at the familiar one only when constituent elements of the rule were upright faces but not in the case of inverted faces. Error bars represent standard error of the means. ^∗^*p* < 0.05.

A repeated measures ANOVA was performed on looking times toward test stimuli, with Presentation (First, Second, Third) and Novelty (New, Familiar) as within-subjects factors and Orientation (Upright, Inverted) and Habituation sequence (ABA, ABB) as between-subjects factors. The analysis revealed a main effect of Presentation, *F*(2,134) = 6.95, *p* = 0.010, $\eta_{p}^{2}$ = 0.09, and an interaction between Novelty and Orientation, *F*(2,134) = 6.951, *p* = 0.010, $\eta_{p}^{2}$ = 0.09. As for the main effect of Presentation, infants’ looking times were greater in the first (*M* = 10.28 s, *SD* = 7.8) than in the second presentation (*M* = 8.17 s, *SD* = 5.4) of the test trials, *t*(70) = 2.27, *p* = 0.027, Cohen’s *d* = 0.31, and in the first than in the third presentation (*M* = 7.60 s, *SD* = 4.6) of the test trials, *t*(70) = 3.048, *p* = 0.003, Cohen’s *d* = 0.41. This result might indicate a weariness effect in the last test trials. As for the Novelty × Orientation interaction, when infants were habituated to upright faces, in both habituation conditions (ABB and ABA) they looked more toward the novel (*M* = 11 s, *SD* = 6.7) than to the familiar sequence (*M* = 8.7 s, *SD* = 5.6), *t*(34) = 2.69, *p* = 0.011, Cohen’s *d* = 0.37 (**Figure 2**). When infants were habituated to inverted faces, in contrast, looking times did not differ between the novel (*M* = 7.1 s, *SD* = 3.5) and the familiar sequence (*M* = 7.9 s, *SD* = 3.6), *t*(35) = 0.99, *p* > 0.3. The Habituation sequence × Novelty interaction was not statistically significant, *F* < 1, *p* > 0.6; there was no reliable difference in novelty preference between infants habituated to the ABB and the ABA sequences.

Results indicate that when infants were habituated to a sequence of upright faces that contained a rule-like pattern (ABB or ABA), they looked longer at the novel rule as compared to the familiar one during the test phase, providing evidence that they were able to extract the rule from the habituation sequence and to generalize it to the new face identities presented during the test phase. Conversely, when infants were habituated with triplets of inverted faces, they did not discriminate the familiar rule from the novel one in the test phase, suggesting that infants were not able to detect and to generalize the rule-like pattern when inverted faces were presented.

## General Discussion

Rule learning is a mechanism that allows infants to detect and generalize rule-like patterns. While it has been first proposed that the ability to extract rules from a sequence of elements might be specific to the linguistic domain (Marcus et al., 1999, 2007), it has more recently pointed out that rule learning is not exclusive to language (e.g., Dawson and Gerken, 2009; Rabagliati et al., 2012). One of the factors that seems to modulate infants’ rule learning abilities is the familiarity with the stimuli, perhaps facilitating the types of comparison necessary to extract a rule (Saffran et al., 2007). On this account, rule learning is preferentially evoked by speech because speech is a highly salient and experienced stimulus for infants.

In the present study we further investigated the role of perceptual expertise on 7 months old infants’ rule learning abilities using sequences of faces, a visual stimulus category that is pervasive in the infant’s environment since birth. After being habituated to sequences of upright faces that contained a rule, infants were able to discriminate and generalize the rule to new face identities. In contrast, when inverted faces were used as elements of the sequences, infants were not able to detect the rule, as revealed by the lack of discrimination between the sequences that contained the familiar rule and the novel rule during the test phase. Face inversion might have disrupted infants’ efficacy in processing face information and, in turn, the advantage in extract the rule found for upright faces. According to Saffran et al. (2007), this finding confirms that 7-month-olds’ sequential rule learning is affected by the perceptual expertise with the material to be learned: experience with upright faces might have enhanced infants’ ability to detect and generalize the rule-like patterns by highlighting similarities between sequences that aid regularity learning.

This outcome is in line with previous evidence regarding 7 months old infants’ rule learning limited capacity when unfamiliar visual stimuli such as geometric shapes were presented (Johnson et al., 2009). In contrast to this previous study with geometrical shapes; however, in which infants were able to learn an ABB rule but not an ABA rule, our data with upside-down faces provide evidence that infants were not able to detect either the ABB and the ABA pattern, suggesting that infants’ rule learning from inverted faces is more fragile than infants’ rule learning from geometric shapes. This difference between inverted faces and geometric shapes could be due to the higher perceptual complexity of the inverted faces, making this type of stimulus harder to process as compared to the simple geometric shapes shown by Johnson et al. (2009). The same difficulties in extracting rules have been found when 7–8-month-olds were presented with unfamiliar sign language-like gestures (Rabagliati et al., 2012), or unfamiliar and sequential auditory stimuli, such as animal sounds, pure tones, notes of different timbre (Marcus et al., 2007), and chords (Dawson and Gerken, 2009).

It is worth noting that our data do not allow us to identify which processes underlie infants’ ability to extract rules for upright-face sequences, and infants’ failure to extract rules from inverted-face sequences. Further research is needed to understand which level of processing, featural, or configural, is involved when infants extract a sequential rule from a face sequence, this factor being crucial in affecting visual category learning (Hammer, 2015). In addition, our study focuses on infants’ ability to extract a rule from different individuals within a single frequently experienced category, leaving unresolved whether infants can extract rule-like patterns from different broad categories to which they have been exposed. The comparison between faces and non-face objects is critical for this purpose, as it has been proposed that face-processing specialization would be the result of general processes devoted to the highly expert identification of within-category exemplars from any object class (e.g., Gauthier and Logothetis, 2000; Tarr and Gauthier, 2000).

Overall, the present study suggests that perceptual experience is crucial in enhancing rule-learning abilities in 7 months old infants, supporting the idea that rule learning might be a domain-general mechanism, instead of a mechanism specific for language acquisition. This claim seems to be confirmed by evidence that newborns are not able to detect rules from a stream of linguistic elements (but possess a general ability to detect perceptual repetitions, Gervain et al., 2008), as well as by evidence that 4-month-olds can learn rules from sequences of non-linguistic auditory elements, such as tones or chords (Dawson and Gerken, 2009). It has been claimed that domain-general cognitive biases and previous learning must be considered as potential sources of constraints on subsequent rule learning abilities (Dawson and Gerken, 2009).

We propose that rule learning abilities might be an emerging property of early biases, such as newborns’ ability to detect perceptual repetitions and newborns’ sensitivity to the statistical structure of a sequence of elements (Bulf et al., 2011). The early sensitivity to statistical information might provide a foundation for the acquisition of more complex relations, perhaps by directing infants’ attention toward potential patterns on the basis of proximity in space and time (Johnson et al., 2009). With development, rule learning might then be tuned to those stimuli to which infants are most frequently exposed in their environment, such as speech and faces, providing an advantage to extract rule-like patterns from these categories of stimuli as compared to those categories of stimuli for which infants have less experience, such as tones or chords. Notably, faces and speech are closely related in infants’ environment: speech sounds come from speaking faces, providing infants with multimodal synchronous stimulation. Therefore, it is also possible to speculate that not only faces and speech *per se* may facilitate infants’ rule learning abilities, but these two stimulus categories may support each other in accounting for their rule learning advantage. This hypothesis is consistent with recent Bayesian proposals of cognitive development (e.g., Gopnik and Tenenbaum, 2007), for which a core feature is what the child brings to the learning task (Newcombe, 2011). For example, infants possess a rich set of learning mechanisms supporting pattern identification, including rule learning, and we have shown that such mechanisms are constrained by stimulus familiarity. Future research should explore which characteristics of the stimuli make a rule easy or hard to learn, as well as whether and how infants’ rule learning abilities develop in early infancy.

## Conflict of Interest Statement

The authors declare that the research was conducted in the absence of any commercial or financial relationships that could be construed as a potential conflict of interest.

## References

[B1] Bulf H. Johnson S. P. Valenza E. (2011). Visual statistical learning in the newborn infant. *Cognition* 121 127–132. 10.1016/j.cognition.2011.06.010 21745660

[B2] Dawson C. Gerken L. (2009). From domain-generality to domain-sensitivity: 4-month-olds learn an abstract repetition rule in music that 7-month-olds do not. *Cognition* 111 378–382. 10.1016/j.cognition.2009.02.010 19338982 PMC2680471

[B3] Diamond R. Carey S. (1986). Why faces are not special: an effect of expertise. *J. Exp. Psychol. Gen.* 115 107–117. 10.1037/0096-3445.115.2.107 2940312

[B4] Di Giorgio E. Leo I. Pascalis O. Simion F. (2012). Is the face-perception system human-specific at birth? *Dev. Psychol.* 48 1083–1090. 10.1037/a0026521 22142186

[B5] Frank M. C. Slemmer J. A. Marcus G. F. Johnson S. P. (2009). Information from multiple modalities helps five-month-olds learn abstract rules. *Dev. Sci.* 12 504–509. 10.1111/j.1467-7687.2008.00794.x 19635078 PMC2718773

[B6] Gallay M. Baudouin J. Y. Durand K. Lemoine C. Lécuyer R. (2006). Qualitative differences in the exploration of upright and upside-down faces in four-month-old infants: an eye-movement study. *Child Dev.* 77 984–996. 10.1111/j.1467-8624.2006.00914.x 16942501

[B7] Gauthier I. Logothetis N. K. (2000). Is face recognition not so unique after all? *Cogn. Neuropsychol.* 17 125–142. 10.1080/026432900380535 20945176

[B8] Gauthier I. Nelson C. A. (2001). The development of face expertise. *Curr. Opin. Neurobiol.* 11 219–224. 10.1016/S0959-4388(00)00200-2 11301243

[B9] Gervain J. Macagno F. Cogoi S. Peña M. Mehler J. (2008). The neonate brain detects speech structure. *Proc. Natl. Acad. Sci. U.S.A.* 105 14222–14227. 10.1073/pnas.0806530105 18768785 PMC2544605

[B10] Gopnik A. Tenenbaum J. B. (2007). Bayesian networks, Bayesian learning and cognitive development. *Dev. Sci.* 10 281–287. 10.1111/j.1467-7687.2007.00584.x 17444969

[B11] Hammer R. (2015). Impact of feature saliency on visual category learning. *Front. Psychol.* 6:451 10.3389/fpsyg.2015.00451 PMC4404734 25954220

[B12] Johnson S. P. Fernandes K. J. Frank M. C. Kirkham N. Marcus G. Rabagliati H. (2009). Abstract rule learning for visual sequences in 8-and 11-month-olds. *Infancy* 14 2–18. 10.1080/15250000802569611 19283080 PMC2654175

[B13] Kelly D. J. Liu S. Lee K. Quinn P. C. Pascalis O. Slater A. M. (2009). Development of the other-race effect during infancy: evidence toward universality? *J. Exp. Child Psychol*. 104 105–114. 10.1016/j.jecp.2009.01.006 19269649 PMC3740564

[B14] Kelly D. J. Quinn P. C. Slater A. M. Lee K. Liezhong G. Pascalis O. (2007). The other-race effect develops during infancy: evidence of perceptual narrowing. *Psychol. Sci.* 18 1084–1089. 10.1111/j.1467-9280.2007.02029.x 18031416 PMC2566514

[B15] Langner O. Dotsch R. Bijlstra G. Wigboldus D. H. Hawk S. T. van Knippenberg A. (2010). Presentation and validation of the Radboud Faces Database. *Cogn. Emot.* 24 1377–1388. 10.1080/02699930903485076

[B16] Macchi Cassia V. Bulf H. Quadrelli E. Proietti V. (2014). Age-related face processing bias in infancy: evidence of perceptual narrowing for adult faces. *Dev. Psychobiol.* 56 238–248. 10.1002/dev.21191 24374735

[B17] Macchi Cassia V. Turati C. Simion F. (2004). Can a non specific bias toward top-heavy patterns explain newborns’ face preference? *Psychol. Sci.* 15 379–383. 10.1111/j.0956-7976.2004.00688.x 15147490

[B18] Marcus G. Fernandes K. Johnson S. (2007). Infant rule learning facilitated by speech. *Psychol. Sci.* 18 387–391. 10.1111/j.1467-9280.2007.01910.x 17576276

[B19] Marcus G. F. Vijayan S. Rao S. B. Vishton P. M. (1999). Rule learning by seven-month-old infants. *Science* 283 77–80. 10.1126/science.283.5398.77 9872745

[B20] McKone E. Crookes K. Jeffery L. Dilks D. D. (2012). A critical review of the development of face recognition: experience is far less important than previously believed. *Cogn. Neuropsychol.* 29 174–212. 10.1080/02643294.2012.660138 22360676

[B21] Newcombe N. S. (2011). What is neoconstructivism? *Child Dev. Perspect.* 5 157–160. 10.1111/j.1750-8606.2011.00180.x

[B22] Pascalis O. de Haan M. Nelson C. A. (2002). Is face processing species-specific during the first year of life? *Science* 296 1321–1323. 10.1126/science.1070223 12016317

[B23] Pascalis O. de Schonen S. (1994). Recognition memory in 3- to 4-day-old human neonates. *Neuroreport* 5 1721–1724. 10.1097/00001756-199409080-00008 7827316

[B24] Rabagliati H. Senghas A. Johnson S. P. Marcus G. F. (2012). Infant rule learning: advantage language, or advantage speech? *PLoS ONE* 7:e40517 10.1371/journal.pone.0040517 PMC3399874 22815756

[B25] Saffran J. R. Pollack S. D. Seibel R. L. Shkolnik A. (2007). Dog is a dog is a dog: infant rule learning is not specific to language. *Cognition* 105 669–680. 10.1016/j.cognition.2006.11.004 17188676 PMC2066190

[B26] Slater A. Morison V. Rose D. (1985). Habituation in the newborns. *Infant Behav. Dev.* 13 183–200.

[B27] Tarr M. J. Gauthier I. (2000). FFA: a flexible fusiform area for subordinate-level visual processing automatized by expertise. *Nat. Neurosci.* 3 764–769. 10.1038/77666 10903568

[B28] Turati C. Bulf H. Simion F. (2008). Newborns’ face recognition over changes in viewpoint. *Cognition* 106 1300–1321. 10.1016/j.cognition.2007.06.005 17674966

[B29] Turati C. Macchi Cassia V. Simion F. Leo I. (2006). Newborns’ face recognition: the role of inner and outer facial features. *Child Dev.* 77 297–311. 10.1111/j.1467-8624.2006.00871.x 16611173

[B30] Turati C. Sangrigoli S. Ruel J. de Schonen S. (2004). Evidence of the face inversion effect in 4-month-old infants. *Infancy* 6 275–297. 10.1207/s15327078in0602_8 33430531

[B31] Valenza E. Simion F. Macchi Cassia V. Umiltà C. (1996). Face preference at birth. *J. Exp. Psychol. Hum. Percept. Perform.* 22 892–903. 10.1037/0096-1523.22.4.892 8756957

[B32] Yin R. K. (1969). Looking at upside-down faces. *J. Exp. Child Psychol.* 81 141–145. 10.1037/h0027474

